# Filovirus RefSeq Entries: Evaluation and Selection of Filovirus Type Variants, Type Sequences, and Names

**DOI:** 10.3390/v6093663

**Published:** 2014-09-26

**Authors:** Jens H. Kuhn, Kristian G. Andersen, Yīmíng Bào, Sina Bavari, Stephan Becker, Richard S. Bennett, Nicholas H. Bergman, Olga Blinkova, Steven Bradfute, J. Rodney Brister, Alexander Bukreyev, Kartik Chandran, Alexander A. Chepurnov, Robert A. Davey, Ralf G. Dietzgen, Norman A. Doggett, Olga Dolnik, John M. Dye, Sven Enterlein, Paul W. Fenimore, Pierre Formenty, Alexander N. Freiberg, Robert F. Garry, Nicole L. Garza, Stephen K. Gire, Jean-Paul Gonzalez, Anthony Griffiths, Christian T. Happi, Lisa E. Hensley, Andrew S. Herbert, Michael C. Hevey, Thomas Hoenen, Anna N. Honko, Georgy M. Ignatyev, Peter B. Jahrling, Joshua C. Johnson, Karl M. Johnson, Jason Kindrachuk, Hans-Dieter Klenk, Gary Kobinger, Tadeusz J. Kochel, Matthew G. Lackemeyer, Daniel F. Lackner, Eric M. Leroy, Mark S. Lever, Elke Mühlberger, Sergey V. Netesov, Gene G. Olinger, Sunday A. Omilabu, Gustavo Palacios, Rekha G. Panchal, Daniel J. Park, Jean L. Patterson, Janusz T. Paweska, Clarence J. Peters, James Pettitt, Louise Pitt, Sheli R. Radoshitzky, Elena I. Ryabchikova, Erica Ollmann Saphire, Pardis C. Sabeti, Rachel Sealfon, Aleksandr M. Shestopalov, Sophie J. Smither, Nancy J. Sullivan, Robert Swanepoel, Ayato Takada, Jonathan S. Towner, Guido van der Groen, Viktor E. Volchkov, Valentina A. Volchkova, Victoria Wahl-Jensen, Travis K. Warren, Kelly L. Warfield, Manfred Weidmann, Stuart T. Nichol

**Affiliations:** 1 Integrated Research Facility at Fort Detrick, National Institute of Allergy and Infectious Diseases, National Institutes of Health, Fort Detrick, Frederick, MD 21702, USA; E-Mails: kuhnjens@mail.nih.gov (J.H.K.); lisa.hensley@nih.gov (L.E.H.); anna.honko@nih.gov (A.N.H.); jahrlingp@niaid.nih.gov (P.B.J.); joshua.johnson@nih.gov (J.C.J.); kindrachuk.kenneth@nih.gov (J.K.); matthew.lackemeyer@nih.gov (M.G.L.); gene.olinger@nih.gov (G.G.O.); james.pettitt@nih.gov (J.P.); 2 FAS Center for Systems Biology, Harvard University, Cambridge, MA 02138, USA; E-Mails: kandersen@oeb.harvard.edu (K.G.A.); sgire@oeb.harvard.edu (S.K.G.); pardis@broadinstitute.org (P.C.S.); 3 Information Engineering Branch, National Center for Biotechnology Information, National Library of Medicine, National Institutes of Health, Bethesda, MD 20894, USA; E-Mails: bao@ncbi.nlm.nih.gov (Y.B.); olga.blinkova@nih.gov (O.B.); jamesbr@ncbi.nlm.nih.gov (J.R.B.);; 4 United States Army Medical Research Institute of Infectious Diseases, Fort Detrick, Frederick, MD 21702, USA; E-Mails: sina.bavari.civ@mail.mil (S.B.); john.m.dye1.civ@mail.mil (J.M.D.); Nicole.l.lackemeyer.ctr@mail.mil (N.L.G.); anderw.s.herbert.ctr@mail.mil (A.S.H.); gustavo.f.palacios.ctr@us.army.mil (G.P.); rekha.g.panchal.civ@mail.mil (R.G.P.); louise.pitt@us.army.mil (L.P.); sheli.r.radoshitzky.ctr@mail.mil (S.R.R.); travis.k.warren.ctr@mail.mil (T.K.W.); 5 Institut für Virologie, Philipps-Universität Marburg, 35043 Marburg, Germany; E-Mails: becker@staff.uni-marburg.de (S.B.); Dolnik@staff.uni-marburg.de (O.D.); klenk@mailer.uni-marburg.de (H.-D.K.); 6 National Biodefense Analysis and Countermeasures Center, Fort Detrick, Frederick, MD 21702, USA; E-Mails: richard.bennett@nbacc.dhs.gov (R.S.B.); nicholas.bergman@nbacc.dhs.gov (N.H.B.); michael.hevey@nbacc.dhs.gov (M.C.H.); tadeusz.kochel@nbacc.dhs.gov (T.J.K.); daniel.lackner@nbacc.dhs.gov (D.F.L.); victoria.jensen@nbacc.dhs.gov (V.W-J.); 7 University of New Mexico, Albuquerque, NM 87131, USA; E-Mails: steven_bradfute@yahoo.com (S.B.); 8 Department of Pathology and Galveston National Laboratory, University of Texas Medical Branch, Galveston, TX 77555, USA; E-Mails: alexander.bukreyev@utmb.edu (A.B.); anfreibe@utmb.edu (A.N.F.); cjpeters@UTMB.EDU (C.J.P.);; 9 Department of Microbiology and Immunology, Albert Einstein College of Medicine, Bronx, NY 10461, USA; E-Mail: kartik.chandran@einstein.yu.edu; 10 Institute of Clinical Immunology, Russian Academy of Science, Siberian Branch, Novosibirsk, Novosibirsk Oblast, Russia, 630091; E-Mail: alexa.che.purnov@gmail.com; 11 Department of Virology and Immunology, Texas Biomedical Research Institute, San Antonio, TX 78227, USA; E-Mails: rdavey@txbiomed.org (R.A.D.); agriffiths@txbiomed.org (A.G.); jpatters@txbiomed.org (J.L.P.); 12 Queensland Alliance for Agriculture and Food Innovation, The University of Queensland, St. Lucia, QLD 4072, Australia; E-Mails: r.dietzgen@uq.edu.au (R.G.D.); 13 Los Alamos National Laboratory, Los Alamos, NM 87545, USA; E-Mails: doggett@lanl.gov (N.A.D.); paulf@lanl.gov (P.W.F.); 14 Integrated BioTherapeutics, Inc., Gaithersburg, MD 20878, USA; E-Mails: sven.enterlein@gmail.com (S.E.); 15 World Health Organization, 1211 Geneva, Switzerland; E-Mail: formentyp@who.int; 16 Metabiota, Inc., San Francisco, CA 94104, USA; E-Mail: jpgonzalez@metabiota.com; 17 Department of Microbiology and Immunology, Tulane University School of Medicine, New Orleans, LA 70112, USA; E-Mail: rfgarry@tulane.edu; 18 Department of Biological Sciences, College of Natural Sciences, and African Centre of Excellence for Genomics of Infectious Diseases, Redeemer’s University, Lagos-Ibadan, Ogun State, Nigeria; E-Mail: chappi@hsph.harvard.edu; 19 Laboratory for Virology, Division of Intramural Research, National Institute for Allergy and Infectious Diseases, National Institutes of Health, Hamilton, MT 59840 USA; E-Mail: thomas.hoenen@nih.gov; 20 Federal State Unitary Company “Microgen Scientific Industrial Company for Immunobiological Medicines”, Ministry of Health of the Russian Federation, Moscow, Russia, 115088; E-Mails: g.m.ignatyev@microgen.ru; 21 Portland, OR 97222, USA; E-Mail: microcaddis@gmail.com; 22 Special Pathogens Program, National Microbiology Laboratory, Public Health Agency of Canada, Winnipeg, Manitoba, R3E 3R2, Canada; E-Mails: gary.kobinger@phac-aspc.gc.ca; 23 Centre International de Recherches Médicales de Franceville, B. P. 769, Franceville, Gabon; E-Mails: eric.leroy@ird.fr; 24 Biomedical Sciences Department, Dstl, Porton Down, Salisbury, Wiltshire SP4 0JQ, UK; E-Mails: mslever@mail.dstl.gov.uk (M.S.L.); SJSMITHER@mail.dstl.gov.uk (S.J.S.); 25 Department of Microbiology and National Emerging Infectious Diseases Laboratory, Boston University School of Medicine, Boston, MA 02118, USA; E-Mails: muehlber@bu.edu (E.M.);; 26 Novosibirsk State University, Novosibirsk, Novosibirsk Region, Russia, 630090; E-Mails: nauka@nsu.ru (S.V.N.); shestopalov2@mail.ru (A.M.S.); 27 Department of Medical Microbiology and Parasitology, College of Medicine of the University of Lagos, Idi-Araba, Private Mail Bag 12003, Lagos, Nigeria; E-Mail: omilabusa@yahoo.com; 28 The Broad Institute, Cambridge, MA 02142, USA; dpark@broadinstitute.org (D.J.P.); 29 Center for Emerging and Zoonotic Diseases, National Institute for Communicable Diseases of the National Health Laboratory Service, Sandringham-Johannesburg 2192, Gauteng, South Africa; E-Mails: januszp@nicd.ac.za; 30 Institute of Chemical Biology and Fundamental Medicine, Siberian Branch of the Russian Academy of Sciences, Novosibirsk, Novosibirsk Region, Russia, 630090; E-Mail: lenryab@yandex.com; 31 Department of Immunology and Microbial Science and The Skaggs Institute for Chemical Biology, The Scripps Research Institute, La Jolla, CA 92037, USA; E-Mail: erica@scripps.edu; 32 Computer Science and Artificial Intelligence Laboratory, Massachusetts Institute of Technology, Cambridge, MA 02139, USA; E-Mail: sealfon@gmail.com; 33 Vaccine Research Center, National Institute of Allergy and Infectious Diseases, National Institutes of Health, Bethesda, MD 20892, USA; E-Mails: njsull@mail.nih.gov; 34 Zoonoses Research Unit, University of Pretoria, Private bag X20 Hatfield, Pretoria 0028, South Africa; E-Mail: bobswanepoel@gmail.com; 35 Division of Global Epidemiology, Hokkaido University Research Center for Zoonosis Control, Kita-ku, Sapporo, Japan; E-Mail: atakada@czc.hokudai.ac.jp; 36 Viral Special Pathogens Branch, Division of High-Consequence Pathogens Pathology, National Center for Emerging and Zoonotic Infectious Diseases, Centers for Disease Control and Prevention, Atlanta, GA 30333, USA; E-Mail: jit8@cdc.gov; 37 Prins Leopold Instituut voor Tropische Geneeskunde, 2000 Antwerp, Belgium; E-Mail: gvdgroen@scarlet.be; 38 Laboratory of Molecular basis of viral pathogenicity, CIRI, Inserm U1111, Université de Lyon, UCB-Lyon-1, Ecole-Normale-Supérieure de Lyon, 69365 Lyon cedex 07, France; E-Mails: viktor.volchkov@inserm.fr (V.E.V.); valentina.volchkova@inserm.fr (V.A.V.); 39 Unither Virology, LLC, Silver Spring, MD 20910, USA; kellylynwarfield@gmail.com; 40 Institute of Aquaculture, University of Stirling FK9 4LA, UK; E-Mails: m.w.weidmann@stir.ac.uk

**Keywords:** Bundibugyo virus, cDNA clone, cuevavirus, Ebola, Ebola virus, ebolavirus, filovirid, *Filoviridae*, filovirus, genome annotation, ICTV, International Committee on Taxonomy of Viruses, Lloviu virus, Marburg virus, marburgvirus, mononegavirad, *Mononegavirales*, mononegavirus, Ravn virus, RefSeq, Reston virus, reverse genetics, Sudan virus, Taï Forest virus, virus classification, virus isolate, virus nomenclature, virus strain, virus taxonomy, virus variant

## Abstract

Sequence determination of complete or coding-complete genomes of viruses is becoming common practice for supporting the work of epidemiologists, ecologists, virologists, and taxonomists. Sequencing duration and costs are rapidly decreasing, sequencing hardware is under modification for use by non-experts, and software is constantly being improved to simplify sequence data management and analysis. Thus, analysis of virus disease outbreaks on the molecular level is now feasible, including characterization of the evolution of individual virus populations in single patients over time. The increasing accumulation of sequencing data creates a management problem for the curators of commonly used sequence databases and an entry retrieval problem for end users. Therefore, utilizing the data to their fullest potential will require setting nomenclature and annotation standards for virus isolates and associated genomic sequences. The National Center for Biotechnology Information’s (NCBI’s) RefSeq is a non-redundant, curated database for reference (or type) nucleotide sequence records that supplies source data to numerous other databases. Building on recently proposed templates for filovirus variant naming [<virus name> (<strain>)/<isolation host-suffix>/<country of sampling>/<year of sampling>/<genetic variant designation>-<isolate designation>], we report consensus decisions from a majority of past and currently active filovirus experts on the eight filovirus type variants and isolates to be represented in RefSeq, their final designations, and their associated sequences.

## 1. Introduction

The National Center for Biotechnology Information (NCBI) RefSeq project was initiated to create a nonredundant and curated set of genomic, transcript, and protein sequence records [1]. Genomic RefSeq records provide a reference nucleotide sequence wherein individual protein coding regions and other sequence features are annotated, using the best available experimental data as a guide. Akin to the labeling of reference specimens as type specimens in other taxonomic schemes, RefSeq reference sequences can be considered type sequences for type viruses.

In the case of virological RefSeq records, each viral species was initially represented by only one genome sequence record, and all other genome records for members of the same species, or for different strains, variants, and isolates of the same member of this species were linked to this record as “genome neighbors” [2]. The rationale behind choosing a particular virus isolate sequence as reference sequence is unclear in most cases and has almost never been published. Annotation of individual RefSeq entries was performed using PubMed-indexed experimental data through NCBI inhouse and individual expert curation, since subspecialty-wide committees or expert groups had not been established.

The process of curating genome sequence data must now be fundamentally reformed, since the number of sequenced viral genomes has increased exponentially over the past decade [3]. Little to no experimental data are available for most new virus genomes, and annotation is often computationally transferred from related genomes or predicted *de novo* [4]. Moreover, the utility of reference genomes has expanded to include use in sequence assembly and pathogen detection pipelines [5,6,7,8,9]. With these changes, the data model has adapted, and multiple RefSeq records can now be maintained for several members of a particular virus species. This approach offers representation of the extant sequence diversity (or genotypes) within a particular species. Also, the approach provides a mechanism to maintain well annotated records from experimentally important laboratory isolates and from less studied isolates from the wild.

## 2. Current Filovirus RefSeq Entries

The mononegaviral family *Filoviridae* includes three genera, *Cuevavirus*, *Ebolavirus*, and *Marburgvirus*. Eight distinct filoviruses are recognized as members of a total of seven species distributed among these three genera (Table 1) [10,11,12,13,14]. 

**Table 1:** Summary of the current filovirus taxonomy endorsed by the 2012-2014 ICTV *Filoviridae* Study Group and accepted by the ICTV.

Current Taxonomy and Nomenclature (Ninth ICTV Report and Updates)
Order *Mononegavirales*
Family *Filoviridae*
Genus *Marburgvirus*
Species *Marburg marburgvirus*
Virus 1: Marburg virus (MARV)
Virus 2: Ravn virus (RAVV)
Genus *Ebolavirus*
Species *Taï Forest ebolavirus*
Virus: Taï Forest virus (TAFV)
Species *Reston ebolavirus*
Virus: Reston virus (RESTV)
Species *Sudan ebolavirus*
Virus: Sudan virus (SUDV)
Species *Zaire ebolavirus*
Virus: Ebola virus (EBOV)
Species *Bundibugyo ebolavirus*
Virus: Bundibugyo virus (BDBV)
Genus *Cuevavirus*
Species *Lloviu cuevavirus*
Virus: Lloviu virus (LLOV)

**Figure 1:**
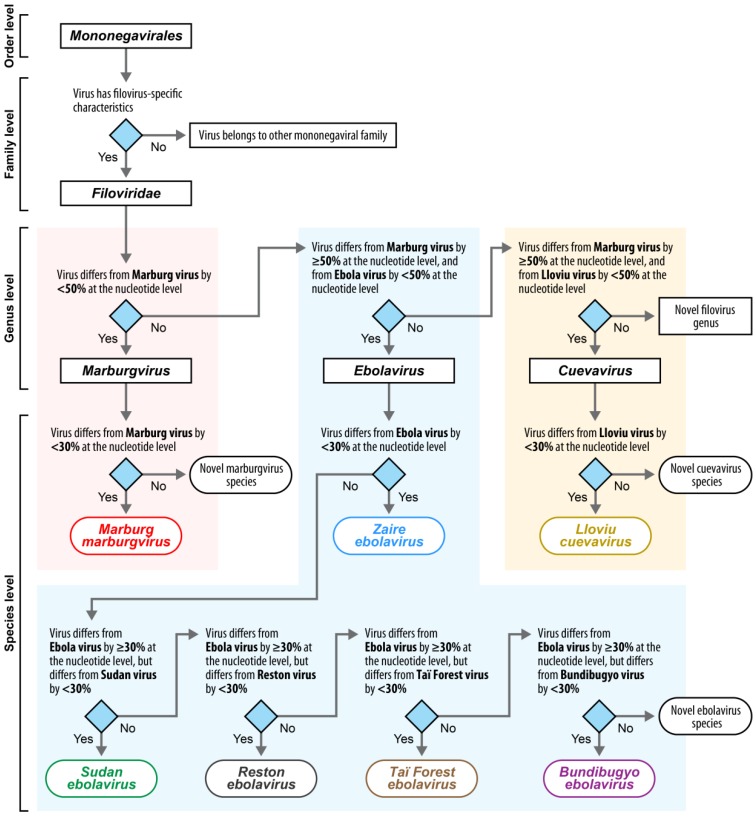
Genome-based classification of novel filoviruses or filovirus genomic sequences. Viruses are classified in the family *Filoviridae* (order *Mononegavirales*) based on a list of biophysical criteria, genomic organization, the type of disease the viruses cause in primates, geographic distribution, and morphology of their virions (outlined in [11,12]). Once a novel virus isolate clearly belongs to this family, genomic sequence comparison can help classification into lower taxa. Novel isolates are classified by comparing the genomic sequence of the new isolate first to the genomic sequences to the type viruses of the ICTV-accepted genera, and then to viruses of the ICTV-accepted species. Using taxon-specific genomic sequence divergence cut-offs, the novel isolate can then be automatically classified into existing taxa, unless they require the establishment of novel taxa through existing ICTV mechanisms.

These eight viruses are differentiated from each other by biological characteristics [12] and genomic sequence divergence [11,12,15,16]. This divergence is determined based on sequences of well-characterized variants of root viruses (from here on called type variants of type viruses) for each taxon [12]. These sequences, therefore, become *de facto* type sequences. Using type sequences allows algorithmic representation of filovirus relationships and newly isolated filoviruses can theoretically be automatically pre-assigned to existing or novel taxa (Figure 1). Temporary type filovirus variants were established by the 2010–2011 ICTV *Filoviridae* Study Group [12]. These temporary type variants were largely consistent with those chosen for RefSeq (Table 2) by the NCBI, which automatically chose the first sequence available for a new virus.

**Table 2:** Temporary filovirus type viruses and type variants chosen by the 2010-2011 ICTV *Filoviridae* Study Group [27], and filovirus type sequences available from RefSeq.

Filovirus Species	Type Virus of Species (Virus Abbreviation)	Type Variant and Isolate of Type Virus of Species	Type Sequence of Type Variant of Type Virus of Species (RefSeq)
*Bundibugyo ebolavirus*	Bundibugyo virus (BDBV)	Unnamed variant represented by isolate “811250”^1^	NC_014373
*Lloviu cuevavirus*	Lloviu virus (LLOV)	Unnamed variant represented by isolate “MS-Liver-86/2003”^2^	NC_016144
*Marburg marburgvirus*	Marburg virus (MARV)	Unnamed variant represented by isolate “Musoke”	NC_001608
*Reston ebolavirus*	Reston virus (RESTV)	Unnamed variant represented by isolate “Pennsylvania”	NC_004161
*Sudan ebolavirus*	Sudan virus (SUDV)	Unnamed variant represented by isolate “Boniface” [sic]^3^	None
*Taï Forest ebolavirus*	Taï Forest virus (TAFV)	Unnamed variant represented by isolate “Côte d’Ivoire”^4^	NC_014372
*Zaire ebolavirus*	Ebola virus (EBOV)	Unnamed variant represented by isolate “Mayinga”	NC_002549

^1^ Isolate “811250” is/was not explicitly mentioned in [12] or RefSeq entry NC_014373 at the time of writing, but could be deduced from [17]; ^2^ The RefSeq isolate name “MS-Liver-86/2003” is mentioned only as “sample 86” in [18]. Note that LLOV has not been isolated in culture yet. “Isolate” here refers to the theoretical isolate, the coding sequences of which would correspond to this RefSeq sequence; ^3^ ”Boneface” is often misspelled “Boniface” in the literature, including in [12]. A review of original sample records at CDC clearly identified the correct name as “Boneface” (Stuart T. Nichol and Pierre E. Rollin, personal communication). RefSeq does not contain a “Boneface” entry but at the time of writing instead listed SUDV variant “Gulu” without an isolate reference (NC_006432); ^4^RefSeq entry NC_014372 did not contain an isolate name at the time of writing.

These variants and sequences therefore needed to be re-evaluated by filovirus experts. To achieve uniformity and consistency, the current RefSeq entries have to be relabeled to conform to current ICTV taxonomy. In addition, type filovirus variant designations have to be chosen and the individual isolate names have to be adjusted to the filovirus strain/variant/isolate schemes that were recently established [19].

## 3. RefSeq Entry Reevaluation

The “gold standard” filovirus type RefSeq entry should be selected on the basis of experimental importance and accessibility and represent a repository of functional information about a particular filovirus. It is of crucial importance that any functional annotation of a RefSeq entry (e.g., functions of particular genome parts or of genome-encoded proteins), is linked to the actual sequence associated with these experiments. The RefSeq entry should contain the most characterized virus/variant/isolate/sequence, independent of whether this virus, variant, or isolate was the first one discovered or the most widely used experimentally. Importantly, decisions on RefSeq entries do not entail a mandate that future experiments should necessarily be performed with the viruses associated with these entries. However, direct comparisons with RefSeq-associated viruses are highly recommended to further increase the detail associated with the RefSeq entries. These entries should be updated, and, if necessary, corrected on a continuous basis by a filovirus RefSeq subcommittee comprised of filovirus experts, whose composition is currently under consideration. 

The authors of this article confirmed or replaced the current taxonomic type virus variants and isolates and the current filovirus RefSeq entries based on the availability of scientific information characterizing a particular virus. If scientific information is scarce for all members belonging to an entire taxon, other criteria such as availability, passaging history, or medical importance were used in decision making. Decisions were reached by consensus or simple majority voting, with the understanding that all authors will apply the final decisions reached by the entire group and enforce them in their functions as authors, peer-reviewers, and/or editors.

### 3.1. Cuevavirus RefSeq Entries

Only one cuevavirus, Lloviu virus (LLOV), has been described [18]. At the time of writing, LLOV had not been isolated in culture, and the sequence diversity of LLOV had only been defined in a single study using deep sequencing techniques on samples from deceased Schreibers’s long-fingered bats (*Miniopterus schreibersii*) [18]. Only one additional study has been published on this virus, characterizing molecular-biological characteristics of the LLOV glycoprotein [20]. The coding-complete genome of one LLOV has been determined (Genbank #JF828358), which therefore automatically became the current RefSeq sequence (#NC_016144) (see [21] for sequencing nomenclature used in this article). In the absence of additional deposited LLOV sequences and characterization data, this RefSeq entry should therefore be upheld but be considered temporary until a complete genome, including all non-coding sequences, is determined.

In line with filovirus strain/variant/isolate definitions outlined previously [19], we propose the variant designation “Asturias” (after the Principality of Asturias in Spain, where Cueva del Lloviu is located in which LLOV was discovered [18]) and the “isolate” name “Bat86” (instead of “MS-Liver-86/2003”) for this virus:
Full name: Lloviu virus M.schreibersii-wt/ESP/2003/Asturias-Bat86Shortened name: LLOV/M.sch/ESP/03/Ast-Bat86Abbreviated name: LLOV/Ast-Bat86

Accordingly, in RefSeq #NC_016144 the definition line “Lloviu virus, complete genome” was changed to “*Lloviu cuevavirus* isolate Lloviu virus M.schreibersii-wt/ESP/2003/Asturias-Bat86, [coding-]complete genome.” The RefSeq <strain> field was cleared; and the RefSeq <isolate> field was filled with “Lloviu virus M.schreibersii-wt/ESP/2003/Asturias-Bat86.” The same changes should be applied to GenBank #JF828358. [Note here and below that the International Nucleotide Sequence Database Collaboration (INSDC) standard currently does not offer options other than “complete” or “partial,” and, in particular, does not provide a possibility for the designation “coding-complete.” Also note here and below that neither RefSeq nor GenBank currently can handle italics or extended Latin characters, which is why the species names are not italicized in the entry’s definition line and <organism> fields and why letters with diacritics revert to their basic Latin letter counterpart].

### 3.2. Ebolavirus RefSeq Entries

The genus *Ebolavirus* includes five species, each of which is represented by one virus.

#### 3.2.1. Bundibugyo Virus

Bundibugyo virus (BDBV) is the second least characterized ebolavirus. Although at least eight isolates of this virus are available [17,22], all experiments reported to date have been performed with one particular isolate, “811250” (often wrongly referred to as “200706291”). The complete sequence of this isolate is the one found in the current RefSeq entry (NC_014373). This isolate, obtained after two passages of clinical material in Vero E6 cells, came from a male patient who died in 2007 in Uganda [17]. We propose the variant designation “Butalya” (after Butalya Parish, Kikyo Subcounty in Uganda’s Bundibugyo district where BDBV was discovered) and the isolate name “811250” for this virus:
Full name: Bundibugyo virus H.sapiens-tc/UGA/2007/Butalya-811250Shortened name: BDBV/H.sap/UGA/07/But-811250Abbreviated name: BDBV/But-811250

Accordingly, in RefSeq #NC_014373, the definition line “Bundibugyo ebolavirus, complete genome” was changed to “*Bundibugyo ebolavirus* isolate Bundibugyo virus H.sapiens-tc/UGA/2007/Butalya-811250, complete genome.” The RefSeq <isolate> field was filled with “Bundibugyo virus H.sapiens-tc/UGA/2007/Butalya-811250.” The same changes should be applied to GenBank #FJ217161.

#### 3.2.2. Ebola Virus

Ebola virus (EBOV) is the most thoroughly characterized ebolavirus. Dozens of EBOV isolates are available, but the vast majority of published experiments have been performed with isolates “Mayinga” and “Kikwit” (reviewed in [23]). The “Mayinga” isolate, the first EBOV isolate obtained in 1976, has been used extensively for molecular-biological characterizations. The “Kikwit” variant, obtained during an Ebola virus disease outbreak in 1995, has been used almost exclusively for pathogenesis studies in nonhuman primates in the US (the “Mayinga” isolate is used almost everywhere else) [23]. All available EBOV cDNA clone systems are based on the “Mayinga” isolate (see [24]). The only available mouse- and guinea pig-adapted EBOV strains are derived from the “Mayinga” isolate (see [25]), and all available EBOV protein crystal structures are derived from the “Mayinga” isolate [26,27,28,29,30,31,32,33,34]. The “Mayinga” isolate was therefore chosen as the prototype EBOV for RefSeq (#NC_002549), which lists a complete genome obtained after 3–4 passages in Vero E6 cells. We uphold this decision and propose the variant designation “Yambuku” (after the village in which EBOV first emerged [35,36]) and retain the isolate designation “Mayinga” (the last name of a nurse who succumbed to infection [36]) for this virus:
Full name: Ebola virus H.sapiens-tc/COD/1976/Yambuku-MayingaShortened name: EBOV/H.sap/COD/76/Yam-MayAbbreviated name: EBOV/Yam-May

Accordingly, in RefSeq #NC_002549 the definition line “Zaire ebolavirus, complete genome” was changed to “*Zaire ebolavirus* isolate Ebola virus H.sapiens-tc/COD/1976/Yambuku-Mayinga, complete genome.” The RefSeq <strain” field was cleared; the RefSeq <isolate> field was filled with “Ebola virus H.sapiens-tc/COD/1976/Yambuku-Mayinga;” and the <organism> field was corrected to “*Zaire ebolavirus*.” The same changes should be applied to GenBank #AF086833.

#### 3.2.3. Reston Virus

Reston virus (RESTV) has caused multiple epizootics among captive macaques (1989-1990, 1992, 1996) and domestic pigs in 2008 (reviewed in [37]). At least 10 isolates were obtained during all these outbreaks, and eight complete or coding-complete genomic sequences have been deposited. However, the vast majority of RESTV experiments, in particular those regarding molecular characterization, have been performed with “Pennsylvania” (reviewed in [23]). “Pennsylvania” is the only RESTV variant for which there is a reverse genetics system [38]. In addition, “Pennsylvania” sequences served as the basis for the available RESTV protein crystal structures [39,40,41,42,43]. “Pennsylvania” (NC_004161) was chosen for the current RESTV RefSeq entry, which we propose to maintain. We propose the variant designation “Philippines89” (a reference to the time and place from which this virus was exported to the US in 1989) and the isolate name “Pennsylvania” for this virus:
Full name: Reston virus M.fascicularis-tc/USA/1989/Philippines89-PennsylvaniaShortened name: RESTV/M.fas/USA/89/Phi89-PenAbbreviated name: RESTV/Phi89-Pen

Accordingly, in RefSeq #NC_004161, the definition line “Reston ebolavirus, complete genome” was changed to “*Reston ebolavirus* isolate Reston virus M.fascicularis-tc/USA/1989/Philippines89-Pennsylvania, complete genome.” The RefSeq <strain> field was cleared; and the RefSeq <isolate> field was filled with “Reston virus M.fascicularis-tc/USA/1989/Philippines89-Pennsylvania”. The same changes should be applied to GenBank #AF522874.

#### 3.2.4. Sudan Virus

Sudan virus (SUDV) is the second-best characterized ebolavirus. Approximately 15 SUDV isolates have been described, but very few experiments have been performed with any of these isolates. Early experiments focused on isolate “Boneface” (often misspelled “Boniface”). Recently variant “Gulu” isolate “808892” has become a more popular choice, and data from experiments with this virus continue to accumulate (reviewed in [23]). Crystal structures for GP_1,2_ were determined for both viruses [33,42,44,45]. However, the passaging history of the “Boneface” isolate has not been thoroughly documented and includes passaging in guinea pigs and culturing in various cell types. The “Gulu-808892” isolate, on the other hand, is completely sequenced and is the current virus of choice for nonhuman primate experiments in the US. While the “Boneface” isolate was chosen by the 2010-2011 ICTV *Filoviridae* Study Group as the type SUDV [12], “Gulu-808892” isolate was chosen as the prototype SUDV for RefSeq (#NC_006432). We propose to support the RefSeq decision and to change the SUDV type virus variant to “Gulu.” As several “Gulu” isolates are available, we propose the variant designation “Gulu” for the virus variant that caused the disease outbreak that started in Gulu District, Uganda, in 2000, and the isolate designation “808892” for the RefSeq entry of this particular virus. (“808892” was obtained after three Vero E6 cell passages of clinical material coming from an infected male who died):
Full name: Sudan virus H.sapiens-tc/UGA/2000/Gulu-808892Shortened name: SUDV/H.sap/UGA/00/Gul-808892Abbreviated name: SUDV/Gul-808892

Accordingly, in RefSeq #NC_006432, the definition line “Sudan ebolavirus, complete genome” was changed to “*Sudan ebolavirus* isolate Sudan virus H.sapiens-tc/UGA/2000/Gulu-808892, complete genome.” The RefSeq <strain> field was cleared; and the RefSeq <isolate> field was filled with “Sudan virus H.sapiens-tc/UGA/2000/Gulu-808892.” The same changes should be applied to GenBank #AY729654.

#### 3.2.5. Taï Forest Virus

Taï Forest virus (TAFV) is the least characterized ebolavirus. Only one isolate (“807212” = “CI”) was obtained from a female survivor [46] after seven passages in Vero E6 cells, and the coding-complete genome of this isolate is the only genomic TAFV sequence available [17]. Therefore, this sequence automatically became the current RefSeq sequence (#NC_014372). In the absence of additional deposited TAFV sequences and characterization data, this RefSeq entry should therefore be upheld but be considered temporary.

We propose the variant designation “Pauléoula” (after the village of Pauléoula, Guiglo Department in Moyen-Cavally Region, Côte d’Ivoire, where TAFV was first found [46]) and the isolate name “CI” (for “Côte d’Ivoire”) for this virus:
Full name: Taï Forest virus H.sapiens-tc/CIV/1994/Pauléoula-CIShortened name: TAFV/H.sap/CIV/94/Pau-CIAbbreviated name: TAFV/Pau-CI

Accordingly, in RefSeq # NC_014372, the definition line “Tai Forest ebolavirus, complete genome” was changed to “*Taï Forest ebolavirus* isolate Taï Forest virus H.sapiens-tc/CIV/1994/Pauléoula-CI, [coding-]complete genome,” and the RefSeq <isolate> field was filled with “Taï Forest virus H.sapiens-tc/CIV/1994/Pauléoula-CI.” The same changes should be applied to GenBank #FJ217162.

### 3.3. Marburgvirus RefSeq Entries

The genus *Marburgvirus* includes a single species, which is represented by two divergent viruses.

#### 3.3.1. Marburg Virus

Marburg virus (MARV) is the most thoroughly characterized marburgvirus. Some 70 MARV isolates are available, but the majority of published experiments have been performed with isolate “Musoke” (reviewed in [23]). However, experiments not characterizing MARV but rather the disease it causes are increasingly performed with an “Angola” isolate in the US and continue to be performed with “Popp” or “Voege” isolates in Russia. The only available MARV cDNA clone systems are based on the “Musoke” isolate (see [24]) and on a nonhuman (bat) isolate [47]. The “Musoke” isolate has therefore been chosen as the prototype MARV for RefSeq (#NC_001608). We uphold this decision and propose the variant designation “Mt. Elgon” (after Mount Elgon, Kenya, where this variant is thought to have originated [48]) and the isolate designation “Musoke” (after a Nairobi doctor who got infected [49]) with this virus):
Full name: Marburg virus H.sapiens-tc/KEN/1980/Mt. Elgon-MusokeShortened name: MARV/Hsap/KEN/80/MtE-MusAbbreviated name: MARV/MtE-Mus

Accordingly, in RefSeq #NC_001608, the definition line “Marburg marburgvirus, complete genome” was changed to “*Marburg marburgvirus* isolate Marburg virus H.sapiens-tc/KEN/1980/Mt. Elgon-Musoke, complete genome.” The RefSeq <strain” field was cleared, and the RefSeq <isolate> field was filled with “Marburg virus H.sapiens-tc/KEN/1980/Mt. Elgon-Musoke.” The same changes should be applied to GenBank #DQ217792.

## 3.3.2. Ravn Virus

Ravn virus (RAVV) is a largely uncharacterized marburgvirus that belongs to the same species as MARV. At least three human (“Ravn” = “810040,” “09DCR,” ”02Uga”) and four Egyptian rousette isolates (“44Bat,” “188Bat,” “982Bat,” “1304 Bat”) have been obtained. Virtually all RAVV characterization experiments have been performed with “Ravn” = “810040,” which was obtained after at least two passages in SW-13 cells and four passages in Vero E6 cells. Since RAVV is a phylogenetically distinct marburgvirus, we created a RefSeq entry for the “Ravn” isolate, for which we propose the variant designation “Kitum Cave” (after Kenya’s Kitum Cave on Mount Elgon where RAVV first emerged) and the isolate designation “810040”:
Full name: Ravn virus H.sapiens-tc/KEN/1987/Kitum Cave-810040Shortened name: RAVV/H.sap/KEN/87/KiC-810040Abbreviated name: RAVV/KiC-810040

Accordingly, the RefSeq entry was created with the definition line “*Marburg marburgvirus* isolate Ravn virus H.sapiens-tc/KEN/1987/Kitum Cave-810040, [coding-]complete genome.” The RefSeq <isolate> field contains “Ravn virus H.sapiens-tc/KEN/1987/Kitum Cave-810040.” The deposited sequence (NC_024781) is identical with GenBank #DQ447649, which should be updated accordingly.

A summary of the proposed designations and RefSeq accession numbers can be found in Table 3.

**Table 3:** Final filovirus type viruses/variants/isolates/sequences.

Filovirus Species	Type Virus of Species (Virus Abbreviation)	Type Variant and Isolate of Type Virus of Species	Type Sequence of Type Variant of Type Virus of Species (RefSeq)
*Bundibugyo ebolavirus*	Bundibugyo virus (BDBV)	Bundibugyo virus H.sapiens-tc/UGA/2007/Butalya-811250	NC_014373
*Lloviu cuevavirus*	Lloviu virus (LLOV)	Lloviu virus M.schreibersii-wt/ESP/2003/Asturias-Bat86^1^	NC_016144
*Marburg marburgvirus*	Marburg virus (MARV)	Marburg virus H.sapiens-tc/KEN/1980/Mt. Elgon-Musoke	NC_001608
*Reston ebolavirus*	Reston virus (RESTV)	Reston virus M.fascicularis-tc/USA/1989/Philippines89-Pennsylvania	NC_004161
*Sudan ebolavirus*	Sudan virus (SUDV)	Sudan virus H.sapiens-tc/UGA/2000/Gulu-808892	NC_006432
*Taï Forest ebolavirus*	Taï Forest virus (TAFV)	Taï Forest virus H.sapiens-tc/CIV/1994/Pauléoula-CI	NC_014372
*Zaire ebolavirus*	Ebola virus (EBOV)	Ebola virus H.sapiens-tc/COD/1976/Yambuku-Mayinga	NC_002549

^1^ Note that LLOV has not been isolated in culture yet. “Isolate” here refers to the theoretical isolate, the coding sequences of which would correspond to this RefSeq sequence.
